# An autotransporter display platform for the development of multivalent recombinant bacterial vector vaccines

**DOI:** 10.1186/s12934-014-0162-8

**Published:** 2014-11-25

**Authors:** Wouter SP Jong, Maria H Daleke-Schermerhorn, David Vikström, Corinne M ten Hagen-Jongman, Karin de Punder, Nicole N van der Wel, Carolien E van de Sandt, Guus F Rimmelzwaan, Frank Follmann, Else Marie Agger, Peter Andersen, Jan-Willem de Gier, Joen Luirink

**Affiliations:** Department of Molecular Cell Biology, Section Molecular Microbiology, Faculty of Earth and Life Sciences, VU University, De Boelelaan 1085, 1081 HV Amsterdam, The Netherlands; Abera Bioscience AB, SE-111 45 Stockholm, Sweden; Xbrane Bioscience AB, SE-111 45 Stockholm, Sweden; Department of Biochemistry and Biophysics, Center for Biomembrane Research, Stockholm University, SE-106 91 Stockholm, Sweden; The Netherlands Cancer Institute, Antoni van Leeuwenhoek Hospital, 1066 CX Amsterdam, The Netherlands; Department of Viroscience, Erasmus Medical Center, 3015 GE Rotterdam, The Netherlands; Department of Infectious Disease & Immunology, Statens Serum Institut, Copenhagen, Denmark; Present Address: Institute for Medical Psychology, Charité Universitätsmedizin, 10117 Berlin, Germany; Present Address: Department of Cell Biology and Histology, Academic Medical Center, University of Amsterdam, 1105 AZ Amsterdam, The Netherlands

**Keywords:** Antigen delivery, Recombinant live vaccine, Surface display, Autotransporter, Multivalent

## Abstract

**Background:**

The Autotransporter pathway, ubiquitous in Gram-negative bacteria, allows the efficient secretion of large passenger proteins via a relatively simple mechanism. Capitalizing on its crystal structure, we have engineered the *Escherichia coli* autotransporter Hemoglobin protease (Hbp) into a versatile platform for secretion and surface display of multiple heterologous proteins in one carrier molecule.

**Results:**

As proof-of-concept, we demonstrate efficient secretion and high-density display of the sizeable *Mycobacterium tuberculosis* antigens ESAT6, Ag85B and Rv2660c in *E. coli* simultaneously. Furthermore, we show stable multivalent display of these antigens in an attenuated *Salmonella* Typhimurium strain upon chromosomal integration. To emphasize the versatility of the Hbp platform, we also demonstrate efficient expression of multiple sizeable antigenic fragments from *Chlamydia trachomatis* and the influenza A virus at the *Salmonella* cell surface.

**Conclusions:**

The successful efficient cell surface display of multiple antigens from various pathogenic organisms highlights the potential of Hbp as a universal platform for the development of multivalent recombinant bacterial vector vaccines.

**Electronic supplementary material:**

The online version of this article (doi:10.1186/s12934-014-0162-8) contains supplementary material, which is available to authorized users.

## Introduction

Live attenuated strains of pathogenic bacteria that synthesize heterologous antigens are being developed as vaccines for several infectious diseases and cancer. Attenuated derivatives of *Salmonella enterica* serovar Typhimurium, a facultative intracellular bacterium capable of provoking strong mucosal and systemic cellular immune responses, have been most extensively studied for this purpose [1]. Using *Salmonella* vaccine strains, cell surface display or secretion of heterologous antigens has been shown to yield superior immune responses compared to intracellular expression [2,3]. Unfortunately, in *Salmonella* and other Gram-negative bacteria like *Escherichia coli*, efficient secretion and surface display of heterologous antigens is difficult. This is due to the presence of a complex, multi-layered envelope that consists of two membranes (inner and outer) separated by the periplasm that comprises a mesh-like peptidoglycan layer.

The Autotransporter pathway [4,5], also known as the Type Va secretion system [6], represents a ubiquitous and simple mechanism for protein translocation across the Gram-negative cell envelope and is typically used for the secretion of large virulence factors. Autotransporters are organized in three domains [7]: (i) an N-terminal signal peptide that targets the protein to the Sec translocon for translocation across the inner membrane, (ii) a secreted passenger domain that carries the effector function, and (iii) a C-terminal β-domain that integrates into the outer membrane (OM) and facilitates translocation of the passenger from the periplasm into the extracellular space [4,5] via a mechanism that also involves the host-derived β-barrel assembly machinery (Bam) complex [8,9]. The Autotransporter system has been used for extracellular expression of antigens, mostly upon direct fusion of heterologous sequences to the β-domain [10]. Although yielding promising results [4,10,11], in the context of vaccine strains like attenuated *Salmonella* these attempts only concerned single antigens or multiple small epitopes. Moreover, reported expression and secretion efficiencies were often low or difficult to evaluate [12-24].

**Figure 1:**
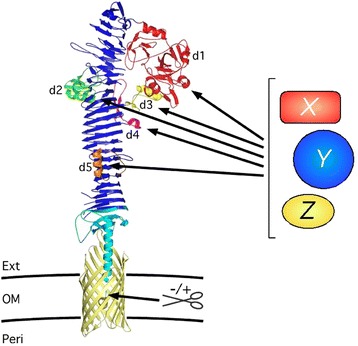
**Strategy for Hbp-mediated secretion and display of heterologous antigens.** Schematic representation of the secretion and display strategy based on the Hbp passenger and β-domain crystal structures [25,27]. Heterologous antigens *x*, *y*, and *z* are fused to the Hbp passenger domain, individually or simultaneously, by (partially) replacing any of the side domains d1 (*red*), d2 (*green*), d3 (*yellow*), d4 (*magenta*) or d5 (*orange*). Scissors indicate a cleavage site between the passenger and β-domain, which was left intact (+) for secretion purposes and disrupted (−) for surface display. The image was created using MacPyMol.

Making use of the crystal structure of its secreted passenger domain [25], we have recently engineered the *E. coli* autotransporter Hemoglobin protease (Hbp) into an efficient platform for the secretion and display of heterologous proteins [15]. The structure features a long (~100 Å) β-helical stem (β-stem) that appears to function as a stable scaffold for five protruding side domains (d1-d5) (Figure 1; Additional file 1: Figure S1) [25]. Whereas the basic β-stem structure is well conserved among autotransporters and has been implicated in autotransporter biogenesis and transport [26], the passenger side domains are dispensable for secretion of Hbp and can be replaced by the *Mycobacterium tuberculosis* antigen ESAT6. Using this strategy, ESAT6 was efficiently transported to the extracellular environment (surface display or secretion) of *E. coli* and attenuated *S*. Typhimurium [15].

Here, we present a systematic analysis to explore whether Hbp can be used as a platform for simultaneous display or secretion of multiple antigenic proteins (Figure 1) to enable the production of multivalent vaccines. As proof of concept, we demonstrate efficient secretion and high-density display of the well-known *Mycobacterium tuberculosis* antigens and vaccine targets ESAT6, Ag85B and Rv2660c [28] incorporated in one Hbp molecule, both in *E. coli* and an attenuated *S.* Typhimurium vaccine strain. Using Hbp as a carrier we also achieved efficient surface exposure of antigenic fragments from the *Chlamydia trachomatis* major outer membrane protein (MOMP) as well as sizeable conserved domains and epitopes from the influenza A virus. These data underline the potential of Hbp as a versatile carrier for high-density surface display of antigens to produce multivalent bacterial live vaccines. It is important to note that this seminal live platform has guided the development of two derived non-living platforms, outer membrane vesicles [29] and bacterial ghosts (De Gier *et al.*, submitted), which can both be decorated with the same Hbp fusion proteins and are considered very safe alternatives for live bacterial vector vaccines [30,31]. The common basis of the three platforms enables the rapid development of vaccine candidates that are tailored to specific requirements.

## Results

### Secretion of the split mycobacterial antigen Ag85B

We have previously shown that the Hbp passenger side domains d1–d5 are dispensable for secretion of Hbp and can be replaced by a small flexible spacer of alternating glycine and serine residues. Furthermore, insertion of the well-known 9.9 kDa *M. tuberculosis* antigen ESAT6 [32] into these linkers to replace any of the side domains d1–d5 resulted in successful secretion of the antigen into the extracellular space [15].

ESAT6 folds into an α-helical hairpin [33], a relatively simple structure that was previously shown to be compatible with Hbp-mediated translocation [9]. To analyze the tolerance of the Hbp system towards more complex antigens, we analyzed the secretion of the Ag85B protein, a secretory 31 kDa T-cell antigen from *M. tuberculosis* with a globular structure containing one disulfide bond [34]. Hbp(Δd1)-Ag85B, carrying the Ag85B moiety at position d1 of the Hbp passenger (Additional file 2: Figure S2), was cloned under *lac*UV5-promoter control into vector pEH3 and expressed in *E. coli* strain MC1061. Cells were grown to early log-phase after which the expression of Hbp was induced by the addition of isopropyl β-D-thiogalactopyranoside (IPTG). Growth was continued and 2 h after induction samples were collected and centrifuged to separate cells and spent medium. To monitor expression and secretion of Hbp, both fractions were analyzed by sodium dodecyl sulfate polyacrylamide gel electrophoresis (SDS-PAGE) and Coomassie staining (Additional file 3: Figure S3A). Unfortunately, Ag85B fused to Hbp appeared to be largely secretion incompetent (lane 5–6, Additional file 3: Figure S3A) and degraded in the periplasm by the protease DegP as evidenced by the vast accumulation of non-processed 146 kDa Hbp-Ag85B pro-form material in cells lacking a proteolytically active periplasmic protease DegP (lane 11, Additional file 3: Figure S3A). The secretion block was only marginally relieved by using a strain that lacks the oxidoreductase DsbA (cf. lane 5–6 and 11–12, Additional file 3: Figure S3B), which is required for the formation of potentially obstructing disulfide bonded loops [35], arguing that mainly other structural restraints prevent secretion.

In an effort to solve this problem, we considered the structure of Ag85B [34] to split it in Ag85B_[N]_ (residues 1–126) and Ag85B_[C]_ (residues 118–285) domains. Ag85B_[N]_ and Ag85B_[C]_ were used to replace domains d1 and d2 using the strategy that has been described above for ESAT6. Both fragments appeared fully compatible with translocation across the cell envelope when inserted individually into the Hbp passenger domain, as judged by the emergence of cleaved passenger and β-domain species upon expression of the corresponding Hbp derivatives in *E. coli* MC1061 (lanes 3–6, Additional file 4: Figure S4). Remarkably, in contrast to Ag85B_[N]_ and ESAT6 [15], fusion to Ag85B_[C]_ affected release of the Hbp passenger into the culture medium (lane 5, Additional file 4: Figure S4). It should be noted that the mechanism of passenger release, or rather retention at the OM, is unknown. Apparently, this process can vary depending on the nature of the inserted heterologous sequences.

**Figure 2:**
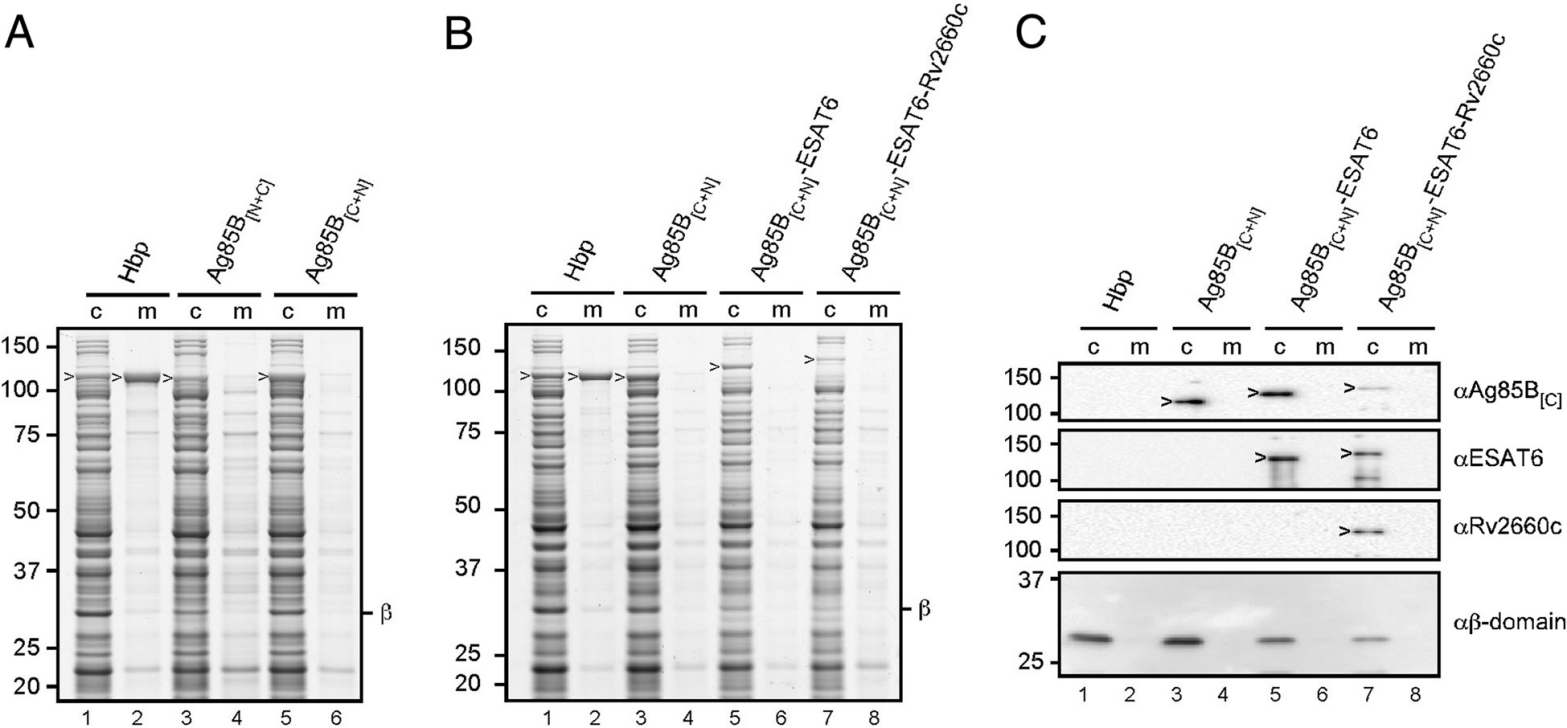
**Secretion of multiple antigens fused to the Hbp autotransporter. (A-B)** Hbp constructs were expressed in *E. coli* MC1061 and the equivalent of 0.03 OD_660_ units cells (c) and corresponding culture medium (m) samples was analyzed by SDS-PAGE and Coomassie staining. **(A)** Expression and secretion of Hbp, Hbp-Ag85B_[N+C]_ and Hbp-Ag85B_[C+N]_. **(B)** Expression and secretion of Hbp, Hbp-Ag85B_[C+N],_ Hbp-Ag85B_[C+N]_-ESAT6 and Hbp-Ag85B_[C+N]_-ESAT6-RV2660c. **(C)** Samples described under *B* were analyzed by immunoblotting using the antibodies indicated. Cleaved Hbp passenger (>) and β-domain (*β*) are indicated. Molecular weight markers (kDa) are shown at the left side of the panels.

The observed extracellular expression of Ag85B_[N]_ and Ag85B_[C]_ encouraged us to combine the two fragments in one Hbp carrier fusing Ag85B_[N]_ at d1 and Ag85B_[C]_ at d2 (Hbp-Ag85B_[N+C]_) and *vice versa* (Hbp-Ag85B_[C+N]_; Additional file 2: Figure S2). The two split Ag85B-Hbp versions were indeed expressed and processed, although release into the medium was affected compared to wild-type Hbp (Figure 2A, cf. lanes 1–2 and 3–6), similar to Hbp only carrying Ag85B_[C]_ (lane 5, Additional file 4: Figure S4). Surprisingly, the positioning of the Ag85B domains in Hbp influenced the efficiency of transport with Hbp-Ag85B_[C+N]_ being more proficient (Figure 2A, cf. lanes 3 and 5). Importantly, the cleaved chimeric passengers were intact as judged by their apparent molecular mass (Figure 2A) and reaction with monoclonal antibodies against the Ag85B_[C]_ moiety (Figure 2C, lane 3). Furthermore, they were fully accessible to Proteinase K, indicating translocation across the OM (Additional file 5: Figure S5A). These data demonstrate efficient simultaneous secretion of Ag85B_[C]_ and Ag85B_[N]_ fused to one Hbp molecule replacing side domains d1 and d2, respectively.

### Secretion and display of multiple mycobacterial antigens

To further explore the heterologous secretion capacity, we added a second (ESAT6), and a third (Rv2660c) [28] *M. tuberculosis* antigen to the existing Hbp-Ag85B_[C+N]_ chimera. ESAT6 was inserted in d4 (Additional file 6: Figure S6; Additional file 2: Figure S2) and d5 was substituted by Rv2660c (Additional file 2: Figure S2). Both positions were shown to be permissive with respect to the insertion of heterologous sequences (Additional file 6: Figure S6) [15]. The resulting 119 kDa Hbp-Ag85B_[C+N]_-ESAT6 and 125 kDa Hbp-Ag85B_[C+N]_-ESAT6-Rv2660c passengers were efficiently transported to the cell surface, and cleaved from their cognate β-domain as judged by their apparent molecular mass and the presence of corresponding amounts of cleaved 28 kDa β-domain in the cell samples (Figure 2B, lanes 5–8; Figure 2C, lanes 5 and 7). Similar to Hbp-Ag85B_[C]_ (lane 5; Additional file 4: Figure S4) and Hbp-Ag85B_[C+N]_ (Figure 2B, lanes 3–4), the passengers were not released to the medium (Figure 2B, lanes 5–8; Figure 2C, lanes 5 and 7). However, their sensitivity to externally added Proteinase K confirmed proper translocation across the OM (Additional file 5: Figure S5). The presence of Ag85B_[C]_, ESAT6 and Rv2660c in the chimeric protein was demonstrated by immunoblotting confirming that the translocated chimeric passengers were intact (Figure 2C). Although substantial amounts of translocated material were observed for all constructs, increasing the number of insertions came at the cost of a gradually lower expression level (Figure 2B). Most likely, the cumulative complexity introduced by the additional insertion of ESAT6 and Rv2660c caused impaired secretion and partial degradation by DegP in the periplasm [35]. Nevertheless, the data clearly show that the Hbp passenger can function as a carrier for efficient secretion of multiple antigens into the extracellular environment.

**Figure 3:**
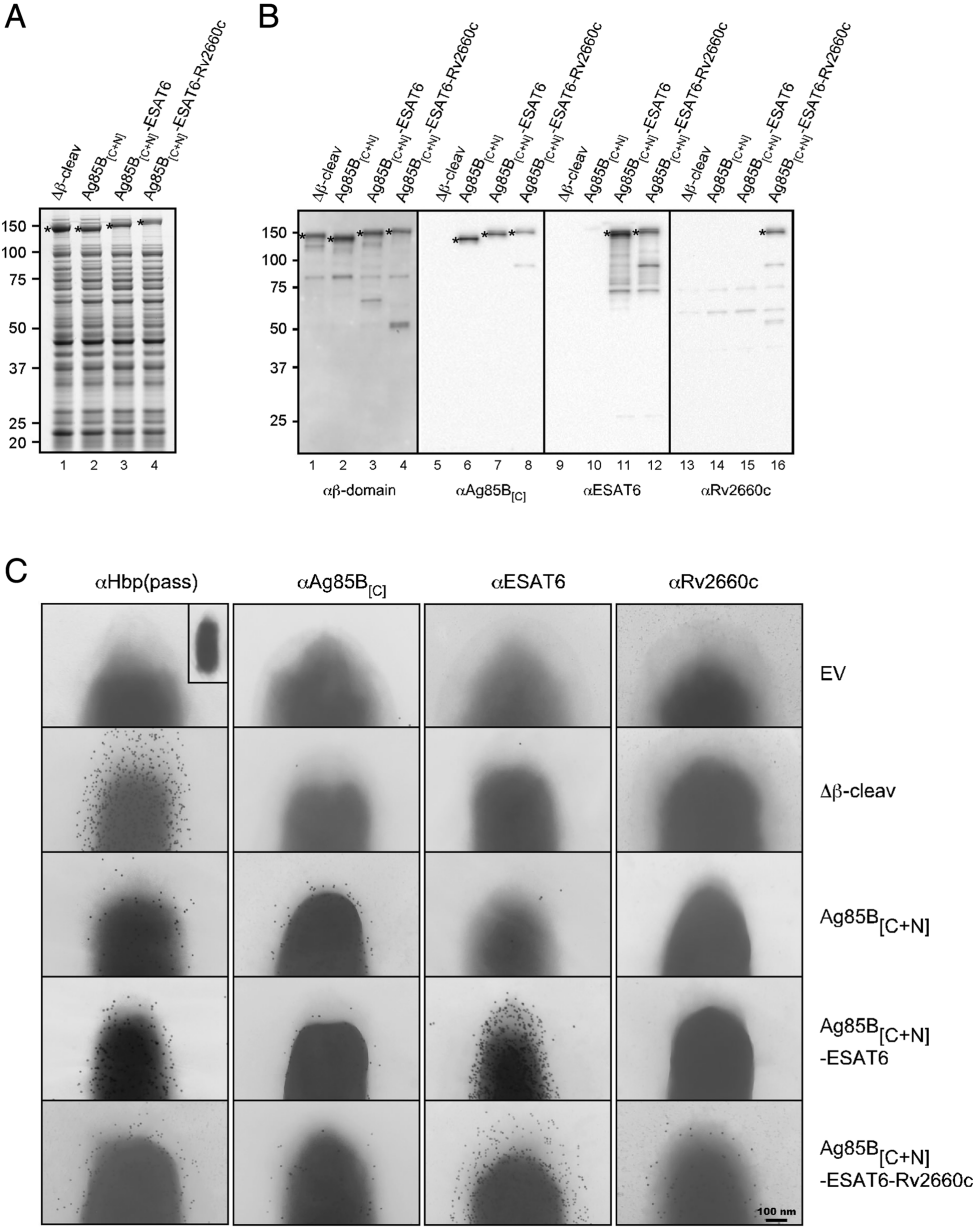
**Display of multiple antigens fused to one Hbp carrier in** ***E. coli*** **. (A-B)** Display of antigens fused to the passenger of the non-cleaved HbpD. *E. coli* MC1061 cells expressing either Hbp(Δβ-cleav), HbpD-Ag85B_[C+N]_, HbpD-Ag85B_[C+N]_-ESAT6 or HbpD-Ag85B_[C+N]_-ESAT6-Rv2660c were analyzed as described in the legend to Figure 2 by Coomassie staining **(A)** and immunoblotting **(B)**. Non-cleaved Hbp species (*) are indicated. **(C)** Cells described under *A* and cells carrying the empty vector (*EV*) pEH3 were fixed and analyzed by immuno-EM using the indicated antibodies as described before [15]. Scale bar: 100 nm.

To enable cell surface exposure of antigens rather than release into the extracellular milieu, we previously constructed a ‘display’ version of the Hbp platform (HbpD) [15] by disrupting the proteolytic cleavage site between the passenger and the β-domain [36]. To test simultaneous display of multiple antigenic proteins, we created the non-cleaved Hbp-antigen chimeras HbpD-Ag85B_[C+N]_, HbpD-Ag85B_[C+N]_-ESAT6 and HbpD-Ag85B_[C+N]_-ESAT6-Rv2660c (Additional file 2: Figure S2). Expression of these constructs was initially analyzed by SDS-PAGE and Coomassie staining (Figure 3A). As expected when the β-domain is not cleaved, the chimeras were detected in the cell fraction with a ~30 kDa increase in apparent molecular mass compared to their cleaved counterparts (cf. Figure 3A and 2B). The presence of the β-domain as well as the Ag85B, ESAT6 and Rv2660c antigens in the respective passenger domains was verified by immunoblotting (Figure 3B). To examine surface exposure of the Hbp passenger and the fused antigens, intact cells were analyzed by immuno-EM using antibodies against the Hbp carrier or the individual antigens (Figure 3C). For all chimeras, clear, dispersed surface labeling was observed using anti-Hbp, demonstrating surface exposure of the respective Hbp passenger domains. Incubation with antibodies against Ag85B_[C]_, ESAT6 or Rv2660c further confirmed display of all antigens at the cell surface (Figure 3C). As a control, cells carrying an empty vector (EV) (Figure 3C) or cells expressing a secretion-incompetent mutant of Hbp (data not shown) were not labeled with any of the antibodies tested. Of note, Hbp constructs lacking the native domain d2 are poorly recognized by anti-Hbp [15], whereas the monoclonal anti-Ag85B_[C]_ recognizes only a small single epitope of Ag85B [37]. This may explain the relatively poor labeling of HbpD-Ag85B_[C+N]_, HbpD-Ag85B_[C+N]_-ESAT6 and HbpD-Ag85B_[C+N]_-ESAT6-Rv2660c expressing cells using these antibodies.

Taken together, using the Hbp platform, efficient and simultaneous extracellular transport was achieved of four heterologous polypeptides, representing three complete mycobacterial antigens.

### Secretion and display of multiple mycobacterial antigens by attenuated *Salmonella* Typhimurium

**Figure 4:**
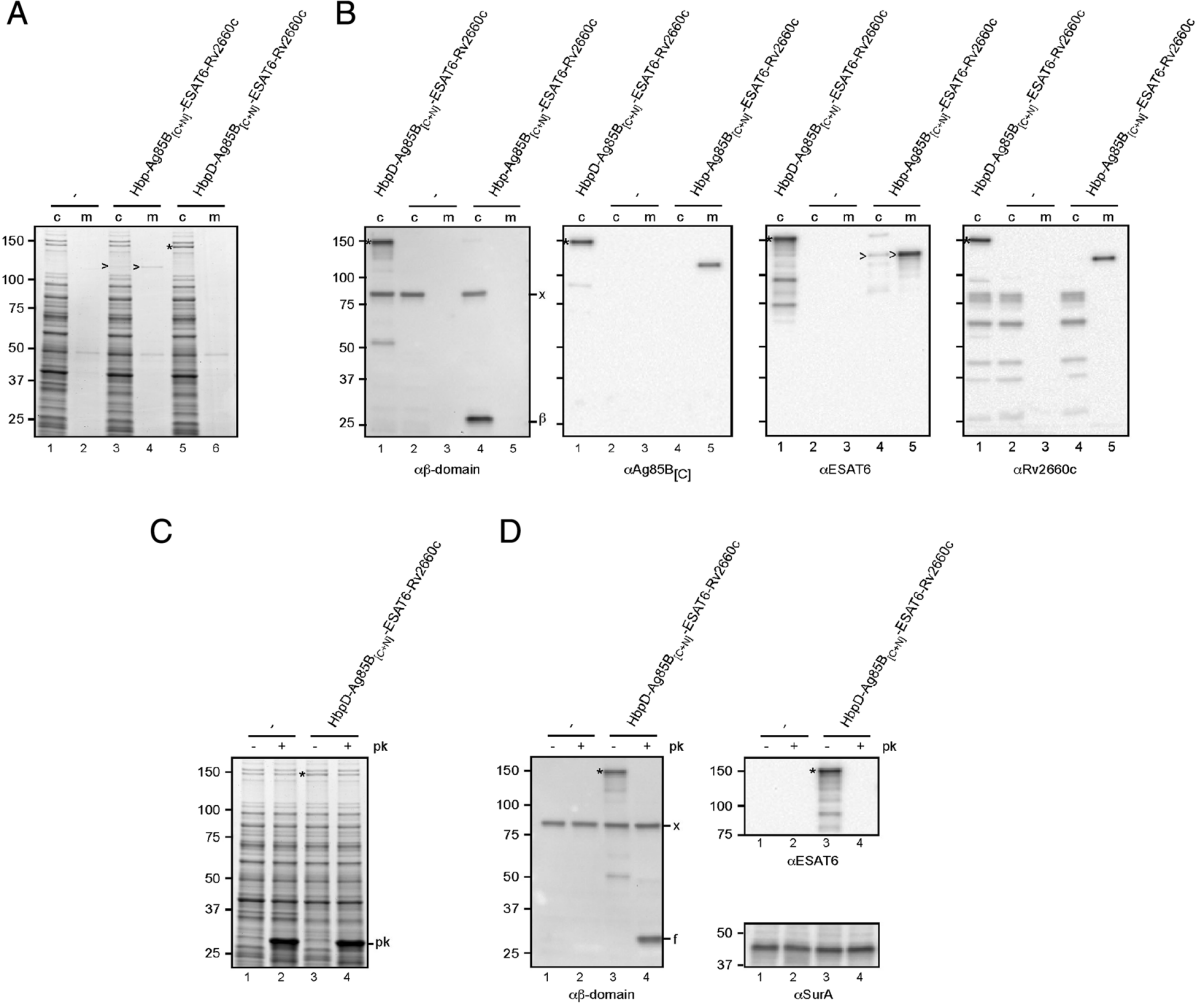
**Secretion and display of antigens by attenuated** ***Salmonella*** **Typhimurium. (A-B)** Secretion and display of antigens fused to the Hbp passenger. *S.* Typhimurium SL3261 (unlabeled) and derivatives expressing Hbp-Ag85B_[C+N]_-ESAT6-Rv2660c or HbpD-Ag85B_[C+N]_-ESAT6-Rv2660c were analyzed by SDS-PAGE and Coomassie staining **(A)** or immunoblotting using the indicated antibodies **(B)**. The equivalent of 0.03 OD_660_ units cells (c) and corresponding culture medium (m) samples was analyzed. **(C-D)** Exposure of antigens at the *S.* Typhimurium cell surface. **(C)** SL3261 cells (lane 1–2) and derivatives expressing HbpD-Ag85B_[C+N]_-ESAT6-Rv2660c from *A* were treated with Proteinase K (+ *pk*) or mock-treated (− *pk*). **(D)** Samples described under *C* were analyzed by immunoblotting. Cell integrity during the procedure was demonstrated by showing the inaccessibility of the periplasmic chaperone SurA towards Proteinase K using anti-SurA (cf. lanes 1, 3, 5 and 2, 4, 6, resp.). Cleaved Hbp passenger (>), non-cleaved Hbp species (*), the cleaved β-domain (*β*) and Proteinase K (*pk*) are indicated. An unrelated protein that cross-reacts with the Hbp β-domain antiserum is indicated (*x*). A proteolytic fragment of HbpD-Ag85B_[C+N]_-ESAT6-Rv2660c is indicated (*f*). Molecular weight markers (kDa) are shown at the left side of the panels.

Attenuated derivatives of *Salmonella enterica* have been proposed as vehicles for the mucosal delivery of heterologous antigens and as a basis for multivalent vaccines [1]. We previously demonstrated Hbp-mediated secretion and display of a single antigen (ESAT6) by the attenuated *S.* Typhimurium SL3261 vaccine strain [15] that has been used for mucosal immunisation in numerous *in vivo* studies [1]. To test Hbp as a platform for the development of multivalent live vaccines, the secretion and display of the antigens ESAT6, Ag85B and Rv2660c was analyzed using *S.* Typhimurium SL3261 as expression host (Figure 4). To achieve stable expression, single copies of the genes encoding either Hbp-Ag85B_[C+N]_-ESAT6-Rv2660c or HbpD-Ag85B_[C+N]_-ESAT6-Rv2660c were integrated into the genome of SL3261. Expression of the genes was controlled by a *lac*UV5 promoter, which is constitutively active in *Salmonella* since this bacterium does not have a *lac* operon and thus does not produce the LacI repressor. Substantial amounts of Hbp passenger containing the three antigens were detected in the culture medium indicating efficient expression, OM translocation and release of Hbp-Ag85B_[C+N]_-ESAT6-Rv2660c in *Salmonella* (Figure 4A, lane 4). Immunoblotting using antibodies specific for Hbp or any of the three mycobacterial antigens confirmed that the chimera was intact (Figure 4B). Of note, when the same Hbp variant was expressed in *E. coli* MC1061, the passenger was almost completely retained at the cell surface (Figure 2B), indicating that the thus far unclear mechanism of passenger release can vary depending on the bacterial expression host used. Conceivably, the smooth and rough lipopolysaccharide phenotypes displayed by *S.* Typhimurium SL3261 [38] and *E. coli* K-12 strain MC1061 [39], respectively, result in differential interactions of the Hbp-Ag85B_[C+N]_-ESAT6-Rv2660c passenger with the bacterial cell surface.

The display variant HbpD-Ag85B_[C+N]_-ESAT6-Rv2660c was detected in the cell fraction of *Salmonella* (Figure 4A, lane 5) in an intact form (Figure 4B). To confirm surface exposure, intact cells expressing the construct were treated with Proteinase K to digest extracellular proteins (Figure 4C). Clearly, HbpD-Ag85B_[C+N]_-ESAT6-Rv2660c was specifically degraded. Maintenance of cell integrity during the procedure was demonstrated by the inaccessibility of the periplasmic chaperone SurA towards Proteinase K (Figure 4D, cf. lanes 3 and 4). Taken together, the Hbp platform allows the efficient extracellular expression of multiple mycobacterial antigens by an attenuated *Salmonella* vaccine strain.

### Display of antigenic sequences from *C. trachomatis* and the influenza virus

We demonstrated effective secretion and display of antigens derived from *M. tuberculosis*. To investigate the versatility of the Hbp system we tested its compatibility with antigenic sequences from two other pathogens: the bacterium *C. trachomatis* and the influenza A virus.

**Figure 5:**
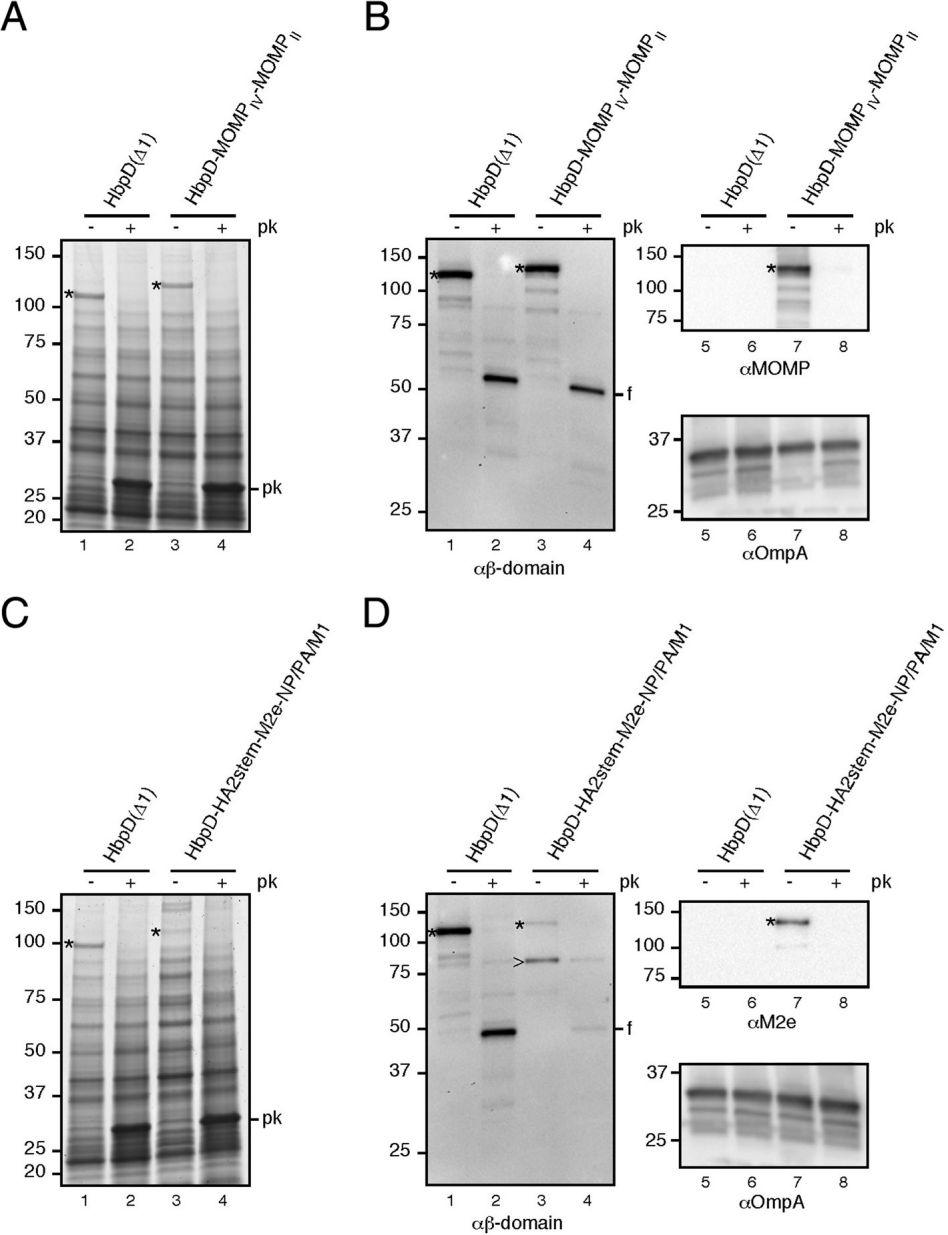
**Display of antigenic fragments from** ***C. trachomatis*** **and the influenza A virus by attenuated** ***Salmonella*** **. (A)** *S*. Typhimurium SL3261 cells expressing HbpD(Δd1) or HbpD-MOMP_IV_-MOMP_II_. Cells were treated with Proteinase K (+ *pk*) or mock-treated (− *pk*) before analysis by Coomassie stained SDS-PAGE. **(B)** Cells from *A* were analyzed by immunoblotting using antibodies against the Hbp β-domain, chlamydial MOMP or OmpA as indicated. **(C)** *S.* Typhimurium SL3261 cells expressing HbpD(Δd1) or HbpD-HA2stem-M2e-NP/PA/M1. Cells were as described under *A*. **(D)** Cells from *C* were analyzed by immunoblotting using antibodies against the Hbp β-domain, influenza M2e and OmpA as indicated. Non-cleaved Hbp species (*), proteolytic fragments of the Hbp-derivatives (*f*) and a truncate of HbpD-HA2stem-M2e-NP/PA/M1 (>) are indicated. Molecular weight markers (kDa) are shown at the left side of the panels.

First we analyzed the Hbp-mediated surface display of sizeable fragments of the immunodominant chlamydial outer membrane protein MOMP (Figure 5A) [40]. We selected a 9 kDa fragment, MOMP_IV_, which corresponds to the predicted surface exposed variable sequence 4 (VS4) region, a cluster of T-cell epitopes located in a predicted periplasmic loop, and a connecting transmembrane β-strand [40]. The MOMP_IV_ sequence was fused to the passenger of HbpD, replacing domain d1. In addition, a 3.4 kDa fragment MOMP_II_ that represents the surface-exposed VS2 region and an adjacent T-cell epitope [40] was inserted into the same HbpD molecule at the position of domain d2 (Additional file 2: Figure S2). Upon production from vector pEH3, the resulting HbpD-MOMP_IV_-MOMP_II_ fusion was expressed with a remarkable efficiency at the surface of *S.* Typhimurium SL3261, almost on par with the non-antigen-carrying control HbpD(Δd1) [15] (Figure 5A, cf. lanes 1 and 3). HbpD-MOMP_IV_-MOMP_II_ was detected by antibodies against the Hbp β-domain or MOMP (Figure 5B, lanes 3 and 7) and displayed a ~10 kDa increase in molecular weight compared to HbpD(Δd1) (Figure 5A, cf. lanes 1 and 3), corroborating the integrity of the construct. Confirming surface localization, the construct appeared accessible to Proteinase K added to intact cells (Figure 5A, lane 4; Figure 5B, lanes 4 and 8) whereas the intracellular Proteinase K-sensitive domain of OmpA [41] remained inaccessible under these conditions (Figure 5B, lane 8).

As an alternative to bacterial antigens, three immunogenic sequences from the influenza A virus were simultaneously fused to HbpD. In this construct, side domain d1 was replaced by a 6.5 kDa fragment of the surface exposed hemagglutinin (HA) 2 protein of influenza A/PR/8/34 that forms a long conserved α–helix in the stem region of the HA [42]. Next, domain d2 was substituted by the first 23 aa of the conserved matrix protein 2 (M2), which constitute the so-called M2 ectodomain (M2e) that is normally exposed at the surface of the influenza virus particle and of infected host cells [43]. Finally, a third sequence encoding a string of immunodominant cytotoxic T cell epitopes [44,45] was inserted into domain d4. This 12 kDa sequence comprised segments of the internal nucleoprotein (NP), the polymerase acidic protein (PA) and matrix protein 1 (M1) from A/PR/8/34, interspaced by short flexible glycine/serine linkers. Upon plasmid-based production in *S.* Typhimurium SL3261, the HbpD-HA2stem-M2e-NP/PA/M1 chimera (Additional file 2: Figure S2) was efficiently expressed at a Coomassie-detectable level (Figure 5C, lane 3) and properly recognized by antibodies against M2e and the β-domain of Hbp (Figure 5D, lanes 3 and 7). Furthermore, in contrast to OmpA (Figure 5D, lane 8), the construct appeared sensitive to incubation with Proteinase K (Figure 5C, lanes 4; Figure 5D, lanes 4 and 8), confirming its localization at the cell surface.

In conclusion, the Hbp platform was successfully used to achieve high-density display of multiple antigenic fragments of bacterial and viral origin at the surface of an attenuated *Salmonella* vaccine strain. These data highlight the potential of Hbp as a versatile generic antigen display platform for the development of multivalent bacterial vector vaccines.

## Discussion

To achieve surface display, antigen fragments have been translationally fused to surface-exposed proteins like integral outer membrane proteins, ice-nucleation protein and fimbriae [46], whereas secretion has been accomplished upon fusion to components of the type I-III secretion pathways [47]. Unfortunately, the size and complexity of the antigens that can be handled by these systems are limited. Many reports indicate that the Autotransporter pathway is better equipped to this task [4,10,11]. However, thus far, efforts to exploit the system for the extracellular expression of antigens in vaccine strains such as attenuated *Salmonella* were restricted to single antigens or multiple small epitopes and yielded limited success [12-24]. Here, we modified the autotransporter Hbp into a multivalent vaccine antigen carrier that can display at least four antigenic sequences from *M. tuberculosis* with a combined mass of ~50 kDa at the cell surface of *E. coli* (Figure 3) and attenuated *S.* Typhimurium (Figure 4). Notably, successful display of this complex chimera could be visualized upon analysis of whole cell material on Coomassie-stained gels (Figure 3) equaling at least ~1.4 × 10^4^ molecules per cell (data not shown) without optimization of expression conditions. In addition, high-density multivalent display was observed of sizeable antigenic fragments from two other pathogens, *Chlamydia trachomatis* and the influenza A virus, emphasizing the flexibility of the Hbp system.

Interestingly, immunization with an Ag85B-ESAT6 fusion protein was previously shown to yield better immune responses and protection against *M. tuberculosis* than a cocktail of the individual antigens, highlighting the benefit of combining multiple antigens in a single Hbp carrier molecule [48,49]. Also, the production of live vaccines consisting of a sole strain exposing multiple antigens is more cost-efficient than formulations comprising a mixture of strains expressing single antigens. Moreover, approaches involving the expression of multiple Hbp-antigen constructs in parallel within a single host may lead to instability at the genetic level due homologous recombination events between the Hbp coding sequences, arguing for the use of a singular translocation system to achieve multivalent antigen display.

The antigens replaced side domains in the Hbp carrier molecule that protrude from the β-stem core in the native structure. We have previously shown that this replacement strategy is critical to maintain the stability of Hbp chimeras upon exposure to the extracellular environment [15]. Furthermore, compared to fusion to truncated autotransporters [10], the intact ~100 Å long β-stem [25] offers the advantage of optimal presentation of antigens at some distance from the cell surface. Although not addressed in this study, the cross-β structure exhibited by the stem of the Hbp passenger has also been suggested to have immunostimulatory properties that are considered beneficial for vaccination purposes [50]. Importantly, replacement of the passenger side-domains by heterologous sequences removes the functional regions of the autotransporter [25,51] with their associated potential toxicity and makes the Hbp platform safe to use for vaccination.

Despite very efficient surface exposure overall, considerable differences in display efficiencies were observed. Whereas HbpD-MOMP_IV_-MOMP_II_ was exported at levels similar to wild-type Hbp, the HbpD-Ag85B_[C+N]_-ESAT6 and HbpD-Ag85B[C+N]-ESAT6-Rv2660c chimeras appeared at the cell surface with reduced efficiencies. One critical parameter seems to be the number of inserted antigens, which appears inversely correlated to the export efficiency of Hbp (Figures 2 and 3). Furthermore, the nature of individual fused sequences may influence the biogenesis of Hbp fusion proteins for example by interfering with proper formation of the β-helical stem [26] or hampering transport via the narrow outer membrane translocation machinery [9]. In the latter case, fusion partners with strong folding potential may compromise translocation, as was observed for full-length Ag85B (Additional file 3: Figure S3). Recent evidence suggests that fused proteins carrying positively charged amino acid stretches affect autotransporter secretion [52]. However, none of the sequences that were inserted into Hbp contained similar positively charged stretches, so charge variation does not explain the differences in display efficiency observed in our study. It should be mentioned that heterologous sequences with a strongly hydrophobic character are not compatible with the Hbp system (data not shown), probably because they cause stalling of the fusion protein already at the level of the Sec-translocon in the inner membrane [53]. Bioinformatics analysis revealed a significant degree of hydrophobicity in Ag85B_[C]_ (data not shown), which may explain the reduced secretion and display efficiencies in constructs carrying this antigen. Interestingly, rather than their features *per se*, the location of individual sequences in the Hbp passenger also plays a role as fusion proteins carrying Ag85B_[N]_ and Ag85B_[C]_ at the d1 and d2 positions, respectively, was less efficiently secreted than its counterpart carrying these domains at the inverse positions, d2 and d1 (Figure 2A).

In line with previous work [9,35], the complex and bulky Ag85B [34] appeared incompatible with Hbp-mediated secretion as a whole and had to be fused as a split antigen in order to sustain secretion via the Hbp pathway. Remarkably, it was recently reported that intact Ag85B can be secreted when fused to a strongly truncated passenger of Pet, a SPATE autotransporter like Hbp [22]. Although in the concerning paper the efficiency of secretion is hard to judge, it is possible that fusion to an intact Hbp passenger domain slows down the secretion kinetics, which could allow Ag85B to fold into a translocation-incompetent conformation. On the other hand, the disparate results may be due to subtle differences in experimental conditions, which can have a significant influence on the secretion of folded proteins via the autotransporter pathway [54].

By using flexible flanking glycine/serine spacer sequences, antigens were fused to the Hbp β-stem in a context that allows their independent movement and folding. It should be noted that native folding of immunizing antigens seems less important for diseases like tuberculosis (TB) that require vaccines that induce cellular immunity [55], which relies on the presentation of extensively processed antigens to the immune system [56]. However, antigen folding may be a critical parameter for eliciting humoral responses to preserve conformational epitopes [57]. In the present work we did not address the conformation of antigens upon fusion to Hbp *per se* but we previously observed Ca^2+^ dependent secretion inhibition of an Hbp-calmodulin fusion protein, indicative of functional folding of calmodulin when fused to Hbp [35]. Furthermore, preliminary data have shown that both monomeric streptavidin [58] and the ZZ domain of protein A from *Staphylococcus aureus* [59] are fully functional in the binding of their ligands biotin and immunoglobulins, respectively, when displayed at the *E. coli* cell surface using Hbp (data not shown). These data demonstrate proper folding of heterologous proteins upon fusion to the Hbp β-stem.

The causative agents of TB, chlamydia and influenza infect individuals via mucosal tissues. Various studies suggest that antigen delivery via the same mucosal routes may elicit local immunity to enhance protection against infection [60]. Attenuated *Salmonella* is regarded as a promising antigen delivery vehicle to meet this purpose as it efficiently invades mucosa-associated lymphoid tissues and provokes strong mucosal as well as systemic immune responses [1]. Importantly, secretion and surface display of antigens has been shown to yield more potent immune responses as compared to expression in the cytoplasm of the vaccine strain [2,3]. Interestingly, extracellular antigen expression induced not only CD4^+^ T cells, as generally observed with antigen delivery by phagocytosed bacteria like *Salmonella*, but also CD8^+^ T cells [2], similar to the delivery of heterologous antigens directly to the cytosol via e.g. the bacterial type III protein secretion system [61]. We have used our Hbp platform to create a live attenuated *Salmonella* strain that displays all constituents of the recently described multistage tuberculosis subunit vaccine H56 (ESAT6-Ag85B-Rv2660c) at the cell surface [28] (Figure 4). In the same context we achieved display of two fragments of the highly immunogenic MOMP that are known to contain important B and T cell epitopes and could form the basis for a vaccine against chlamydial disease [40]. Moreover, two conserved protein fragments plus a string of CD8+ T cell epitopes from the influenza virus, representing promising influenza vaccine targets [43-45,62], were expressed at the surface of *Salmonella*. Whether Hbp-mediated surface expression of abovementioned antigens on live cells or derived outer membrane vesicles and bacterial ghosts will lead to successful vaccination strategies against TB, chlamydia and influenza will be investigated in future challenge studies.

## Conclusions

In the present work we describe the engineering of the autotransporter Hbp into a platform for the secretion or display of multiple recombinant antigens by Gram-negative bacteria. To highlight the capacity and versatility of the platform we demonstrate efficient translocation of up to four sizeable antigenic sequences from various pathogenic organisms (*M. tuberculosis*, *C. trachomatis* and Influenza A virus) per Hbp carrier molecule in *E. coli* and an attenuated *Salmonella* vaccine strain. The Hbp platform can be used for the generation of multivalent recombinant bacterial live vaccines but also for derived non-living vaccines based on outer membrane vesicles or bacterial ghosts.

## Methods

### Strains and culturing conditions

Strain MC1061 [63] was routinely used for expression of Hbp and its derivatives in *E. coli*. Where indicated, *E. coli* strains MC1061*degP::S210A* [64,65], DHB4 [66], DHB4*dsbA*:*:kan* (DHBA) [66] or TOP10F’ (Invitrogen) were used. Plasmid-borne expression of Hbp derivatives in *S*. Typhimurium was carried out using strain SL3261 [67]. To construct *S*. Typhymurium SL3261 strains expressing either Hbp-Ag85B_[C+N]_-ESAT6-Rv2660c or HbpD-Ag85B_[C+N]_-ESAT6-Rv2660c, the respective coding sequences and an upstream *lac*UV5 promoter region were inserted into the chromosome by allelic exchange through double cross-over homologous recombination replacing the *malE* and *malK* promotor regions. This was done as described [15], except that pHbp-Ag85B_[C+N]_-ESAT6-Rv2660c and pHbpD-Ag85B_[C+N]_-ESAT6-Rv2660c (see under *Plasmid construction*) were used as templates to PCR-amplify the sequences for cloning into the suicide vector.

Cells were grown at 37°C in LB medium containing 0.2% glucose. The antibiotics chloramphenicol (30 μg/ml) and streptomycin (25 μg/ml) were added where appropriate.

### Reagents and sera

Restriction enzymes, Alkaline phosphatase and DNA ligase (Rapid DNA Dephos & Ligation Kit), Lumi-light Western blotting substrate and Proteinase K (recombinant, PCR grade) were purchased from Roche Applied Science, Phusion DNA polymerase from Finnzymes, and electron microscopy (EM) grade paraformaldehyde and glutaraldehyde from Electron Microscopy Sciences. The polyclonal antisera against the Hbp passenger (J40) and β-domain (SN477) [68,69], as well as the monoclonal antibodies against ESAT6 (HYB 76–8) and Ag85B_[C]_ (TD17) [37,70] have been described previously. The rabbit polyclonal antisera against OmpA and *C. trachomatis* D/UW-3/CX MOMP, as well as the rat polyclonal antiserum against Rv2660c were from our own lab collection. The rabbit polyclonal antiserum against SurA was a gift from T. Silhavy (Princeton University, USA) and the mouse monoclonal antibody against M2e was a gift from X. Saelens (University of Ghent, Belgium).

### Plasmid construction

All plasmids used are derivatives of pEH3 [71]. pHbp(d4in) was created upon substitution of the coding sequence for residues 708–712 of the passenger of pEH3-Hbp(ΔBamHI) [15] by a Gly/Ser encoding linker sequence containing *Sac*I and *Bam*HI restriction sites using overlap-extension PCR. The primers used were Hbp(d4in) fw and Hbp(d4in) rv, yielding pHbp(d4in).

To insert the coding sequence for ESAT6 into pHbp(d4in), an *E. coli*-codon-usage-optimized synthetic gene of *M. tuberculosis* gene *esxA* was constructed by Baseclear B.V. The synthetic gene was flanked by 5′-gagctcc-3′ and 5′-ggatcc-3′ sequences at the 5′ and 3′ site, respectively, allowing in-frame insertion into the *hbp* ORF using the *Sac*I/*Bam*HI restriction sites, giving rise to pHbp(d4ins)-ESAT6.

Plasmid pHbp-Ag85B was constructed by amplifying the Ag85B-encoding gene *fbpA* with flanking *Sac*I/*Bam*HI restriction sites by PCR using *M. tuberculosis* H37Rv genomic DNA as a template. The primers used were Cas/Ag85B fw and Cas/Ag85B rv. The PCR fragment was cloned into pHbp(Δd1) [15] using the *Sac*I/*Bam*HI restriction sites, resulting in pHbp-Ag85B. To construct pHbp-Ag85B_[N+C]_ and pHbp-Ag85B_[C+N]_, fragments of *fbpA* encoding Ag85B_[N]_ and Ag85B_[C]_ were generated with flanking *Sac*I/*Bam*H sites using *M. tuberculosis* H37Rv genomic DNA as a template. For Ag85B_[N]_, the primers used were Cas/Ag85B fw and Cas/Ag85B(S126) rv. The resulting PCR fragment was cloned into pHbp(Δd1) and pHbp(Δd2) [15] using the *Sac*I/*Bam*HI restriction sites, creating pHbp(Δd1)-Ag85B_[N]_ and pHbp(Δd2)-Ag85B_[N]_, respectively. For Ag85B_[C]_ the primers used were Cas/Ag85B(T118) fw and Cas/Ag85B rv. The resulting PCR fragment was inserted into pHbp(Δd1) and pHbp(Δd2) [15] using the *Sac*I/*Bam*HI restriction sites, creating pHbp(Δd1)-Ag85B_[C]_ and pHbp(Δd2)-Ag85B_[C]_, respectively. Subsequently, the *Xba*I/*Nde*I fragment of pHbp(Δd2)-Ag85B_[C]_ was substituted by the *Xba*I/*Nde*I fragment of pHbp(Δd1)-Ag85B_[N]_, yielding pHbp-Ag85B_[N+C]_, and the *Xba*I/*Nde*I fragment of pHbp(Δd2)-Ag85B_[N]_ was substituted by the *Xba*I/*Nde*I fragment of pHbp(Δd1)-Ag85B_[C],_ giving pHbp-Ag85B_[C+N]_.

To create a plasmid expressing Hbp fused to both Ag85B and ESAT6, the *Nsi*I/*Kpn*I fragment of pHbp-Ag85B_[C+N]_ was substituted by that of pHbp(d4in)-ESAT6, creating pHbp-Ag85B_[C+N]_-ESAT6. To make a version of this plasmid additionally expressing Rv2660c, plasmid pHbp(Δd5)-Rv2660c was created first. To this end, *Rv2660c* with flanking *Sac*I/*Bam*HI sites was amplified by PCR using *M. tuberculosis* H37Rv genomic DNA as a template. The primers used were Cas/Rv2660c fw and Cas/Rv2660c rv. The PCR product was cloned into pHbp(Δd5) [15] using the *Sac*I/*Bam*HI sites, creating pHbp(Δd5)-Rv2660c. Subsequently, the *Bst*Z17i/*Kpn*I fragment of pHbp-Ag85B_[C+N]_-ESAT6 was substituted by that of pHbp(Δd5)-Rv2660c, giving pHbp-Ag85B_[C+N]_-ESAT6-Rv2660c.

To construct display versions of pHbp-Ag85B_[C+N]_, pHbp-Ag85B_[C+N]_-ESAT6 and pHbp-Ag85B_[C+N]_-ESAT6-Rv2660c, the *Xba*I/*Kpn*I fragments of these plasmids were substituted for that of pEH3-HbpD(ΔBamHI) [15]. This resulted in pHbpD-Ag85B_[C+N]_, pHbpD-Ag85B_[C+N]_-ESAT6 and pHbpD-Ag85B_[C+N]_-ESAT6-Rv2660c, respectively.

To create plasmids for expression of epitopes from *C. trachomatis* MOMP, two *E. coli*-codon-optimized synthetic DNA fragments were ordered from Life Technologies that coded for sequences including and flanking the VS2 (‘MOMP_II_’; residues 155–190) and VS4 loops (‘MOMP_IV’_; residues 266–350) of MOMP from *C. trachomatis* D/UW-3/CX. To allow in-frame insertion into the *hbp* ORF of pHbp derivatives by *Sac*I/*Bam*HI digestion, the DNA fragments were synthesized with flanking 5′-gagctcc-3′ and 5′-ggatcc-3′ sequences at the 5′ and 3′ site, respectively. The synthetic sequences were cloned into pEH3-Hbp(Δd2) and pEH3-Hbp(Δd1), respectively [15], yielding pHbp(Δd2)-MOMP_II_ and pHbp(Δd1)-MOMP_IV_. To create a construct for the expression of Hbp fused to both MOMP fragments, the *Nde*I/*Nsi*I fragment of pHbp(Δd1)-MOMP_IV_ was substituted by that of pHbp(Δd2)-MOMP_II_ resulting in pHbp-MOMP_IV_-MOMP_II_. To construct a display version of this construct, the *Kpn*I/*Eco*RI fragment of pHbp-MOMP_IV_-MOMP_II_ was substituted by that of pEH3-HbpD(ΔBamHI), yielding pHbpD-MOMP_IV_-MOMP_II_.

Synthetic *E. coli*-codon-optimized DNA fragments encoding the HA2 stem region of the HA protein of the influenza isolate A/PR/8/34 (H1N1) (aa 76–130) [72], and the universally conserved ectodomain of the influenza M2 protein (aa 1–23) [43] were ordered from Life Technologies. An additional *E. coli*-codon-optimized synthetic DNA sequence was ordered, in which fragments coding for residues 356–401 of the NP, 214–243 of the PA and 48–76 of the M1 proteins of influenza A/PR/8/34, spaced by Gly/Ser-encoding linker sequences, were assembled. The three fragments, ‘HA2stem’, ‘M2e’ and ‘NP/PA/M1’ were flanked by 5′-gagctcc-3′ and 5′-ggatcc-3′ sequences at the 5′ and 3′ site, respectively, allowing insertion into the *hbp* ORF of and pHbpD derivatives by *Sac*I/*Bam*HI digestion. In this way, pHbpD(Δd1)-HA2stem, pHbp(Δd2)-M2e and pHbp(Δd4)-NP/PA/M1 were created. To construct a plasmid for the expression of all three influenza sequences, the *Nsi*I/*Kpn*I fragment of pHbp(Δd2)-M2e was first substituted by that of pHbp(Δd4)-NP/PA/M1, resulting in pHbp-M2e-NP/PA/M1. Subsequently, the *Nde*I/*Kpn*I fragment of this plasmid was substituted for that of pHbpD(Δd1)-HA2stem, resulting in plasmid pHbpD-HA2stem-M2e-NP/PA/M1.

Nucleotide sequences of all constructs were confirmed by semi-automated DNA sequencing. Primer sequences are listed in Table 1.

**Table 1:** **Primers used in this study**

**Primer**	**Sequence (5′ → 3′)**
Hbp(d4in) fw	ctgggagctccgcaggatccggcagcggtaaaagtgtcttcaacggcacc
Hbp(d4in) rv	ctgccggatcctgcggagctcccagaacctgcaacagatgtgccttcttc
Cas/Ag85B fw	cggggagctccttctcccggccggggc
Cas/Ag85B(S126) rv	tgccggatcccgacaagccgattgcagcg
Cas/Ag85B(T118) fw	cggggagctccaccggcagcgctgcaatcg
Cas/Ag85B rv	tgccggatccgccggcgcctaacgaac
Cas/Rv2660c fw	cggggagctccgtgatagcgggcgtcgacc
Cas/Rv2660c rv	tgccggatccgtgaaactggttcaatcccag

### Proteinase K treatment of cells

Cells were resuspended in ice-cold reaction buffer (50 mM Tris HCl, pH 7.4, 1 mM CaCl_2_). Subsequently, Proteinase K was added to a concentration of 100 μg/ml to one half of the suspension, whereas the other half was mock-treated, and the suspensions were incubated at 37°C for 1 h. Thereafter, phenylmethanesulfonyl fluoride (0.1 mM) was added and the suspensions were incubated on ice for 10 min. Samples were then TCA precipitated and analyzed by SDS-PAGE and Coomassie staining or immunoblotting as indicated.

### General protein expression and analysis

For analysis of plasmid-borne expression of Hbp (derivatives), cultures were grown to early log-phase (OD_660_ ≈ 0.3) before protein production was induced by the addition of 1 mM IPTG. Growth was continued for 2 h, after which samples were withdrawn from the cultures for further analysis. For analysis of genome-based expression of Hbp-derivatives, *Salmonella* cultures were grown to mid-log phase before withdrawal of samples. In all cases, culture samples were separated into cells and spent medium by low speed centrifugation, and analyzed by SDS-PAGE followed by Coomassie (G-250) staining or immunoblotting. Cells were resuspended in SDS-sample buffer (125 mM Tris–HCl, pH 6.8, 4% SDS, 20% glycerol, 0.02% bromophenol blue, 100 mM dithiothreitol) directly whereas medium samples were first TCA-precipitated.

## Additional files

Additional file 1: Figure S1. Side domains of the Hbp passenger domain. Additional file 2: Figure S2. Schematic representation of Hbp derivatives used in the study. Additional file 3: Figure S3. Expression of Hbp-Ag85B in *degP*- and *dsbA*-mutant strains. Additional file 4: Figure S4. Secretion of Ag85B_[N]_ and Ag85B_[C]_ upon fusion to Hbp. Additional file 5: Figure S5. Proteinase K accessibility of cleaved Hbp passenger-antigen fusions at the cell surface. Additional file 6: Figure S6. Secretion of ESAT6 inserted into d4.

## Abbreviations

OM: Outer membrane
IPTG: Isopropyl β-D-thiogalactopyranoside
SDS-PAGE: Sodium dodecyl sulfate polyacrylamide gel electrophoresis
TB: Tuberculosis
