# Supplementary material for: An autotransporter display platform for the development of multivalent recombinant bacterial vector vaccines
Source: Microb Cell Fact. 2014 Nov 25;13:162. doi: 10.1186/s12934-014-0162-8 (PMC4252983; doi:10.1186/s12934-014-0162-8)
Supplement: Additional file 1: Figure S1. — Side domains of the Hbp passenger domain. [file 12934_2014_162_MOESM1_ESM.pdf]

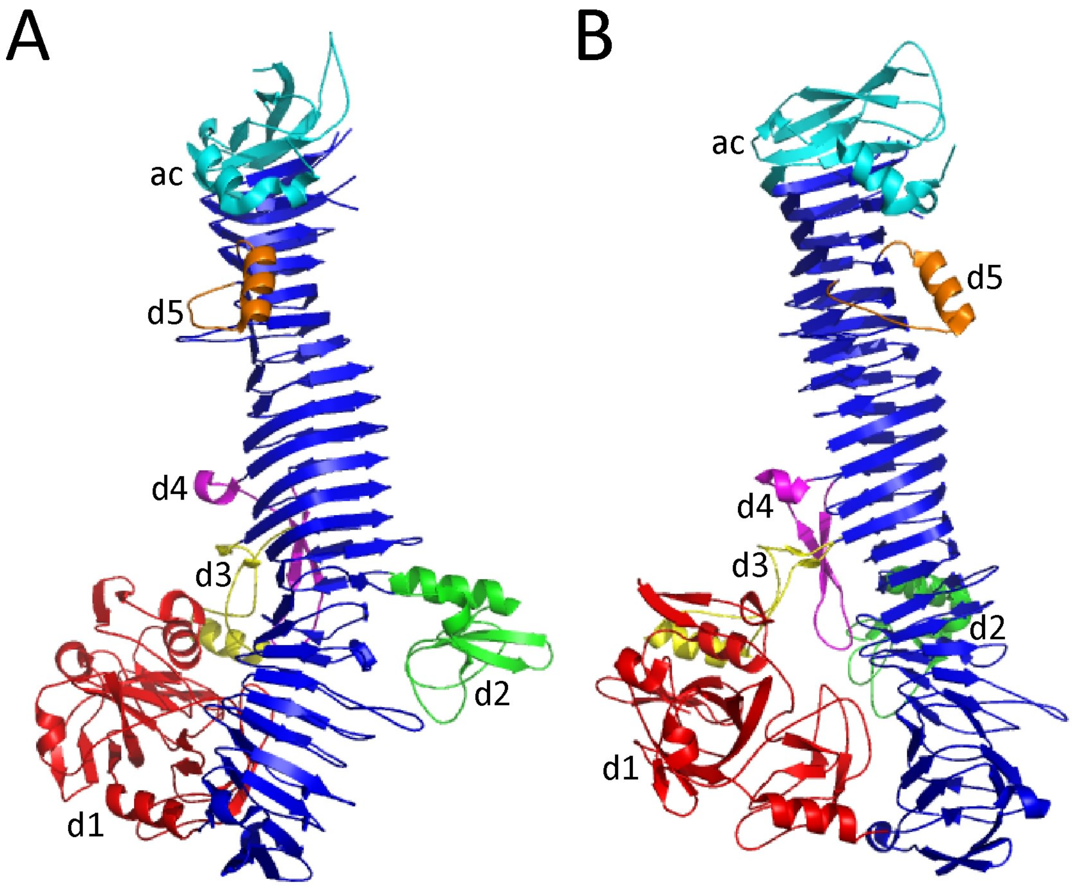

Fig. S1. Side domains of the Hbp passenger domain. (A) Cartoon of the crystal structure of the passenger domain of the *E. coli* AT Hbp (1WXR[PDB]). The side domains d1 (*red*), d2 (*green*), d3 (*yellow*), d4 (*magenta*), d5 (*orange*), and the conserved autochaperone domain (*ac*; *cyan*) are highlighted. The remainder of the passenger, including the β-stem domain, is in dark blue. **(B)** Cartoon of the crystal structure as in *A* rotated around the y-axis (50°counter clockwise). The image was created using MacPyMol.
