# Supplementary material for: An autotransporter display platform for the development of multivalent recombinant bacterial vector vaccines
Source: Microb Cell Fact. 2014 Nov 25;13:162. doi: 10.1186/s12934-014-0162-8 (PMC4252983; doi:10.1186/s12934-014-0162-8)
Supplement: Additional file 2: Figure S2. — Schematic representation of Hbp derivatives used in the study. [file 12934_2014_162_MOESM2_ESM.pdf]

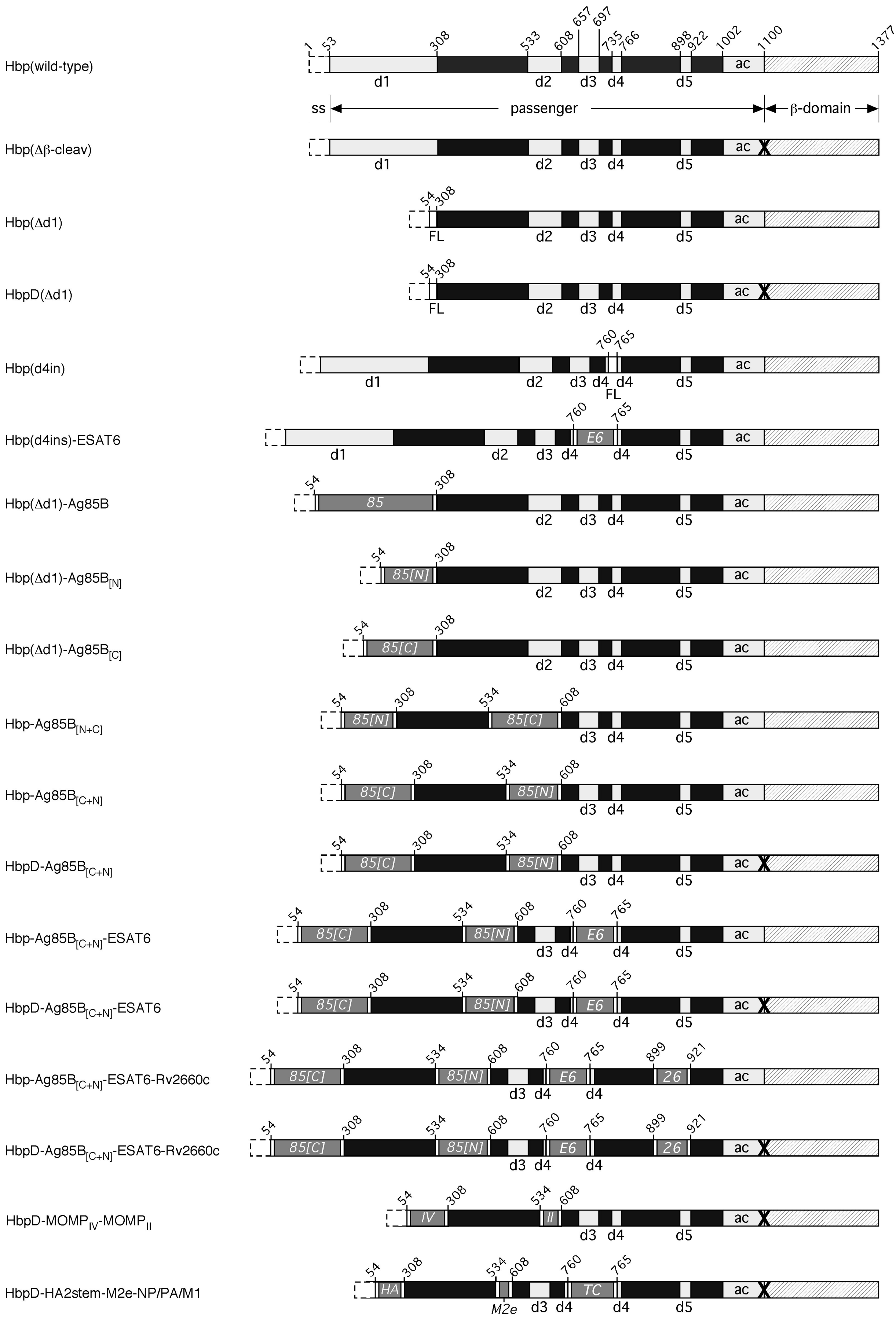

Hbp(wild-type)
1
53
308
533
608
657
697
735
766
898
922
1002
1100
1377
ac
d1
d2
d3
d4
d5
ss
passenger
β-domain
Hbp(Δβ-cleav)
Hbp(Δd1)
54
FL
HbpD(Δd1)
Hbp(d4in)
760
765
Hbp(d4ins)-ESAT6
E6
Hbp(Δd1)-Ag85B
85
Hbp(Δd1)-Ag85B[N]
85[N]
Hbp(Δd1)-Ag85B[C]
85[C]
Hbp-Ag85B[N+C]
534
Hbp-Ag85B[C+N]
HbpD-Ag85B[C+N]
Hbp-Ag85B[C+N]-ESAT6
HbpD-Ag85B[C+N]-ESAT6
Hbp-Ag85B[C+N]-ESAT6-Rv2660c
899
921
26
HbpD-Ag85B[C+N]-ESAT6-Rv2660c
HbpD-MOMP_IV-MOMP_II
IV
II
HbpD-HA2stem-M2e-NP/PA/M1
HA
TC
M2e

Fig. S2. Schematic representation of Hbp derivatives used in the study. Hbp is synthesized as a 1377 amino acid (*aa*) precursor that is organized in three domains: (i) an N-terminal cleavable signal sequence (*ss*; aa 1-52), (ii) a secreted passenger domain (aa 53-1100) and (iii) an outer membrane (OM) integrated C-terminal β-domain (aa 1101-1377). Domain 1 (*d1*), domain 2 (*d2*), domain 3 (*d3*), domain 4 (*d4*), domain 5 (*d5*) and the autochaperone domain (*ac*) of the passenger domain are indicated. The remainder of the passenger domain, including the β-stem is in black. After passage of the OM, the passenger is cleaved from the β-domain via an autocatalytic mechanism that involves hydrolysis of the peptide bond between two asparagines at position 1100 and 1101 of the Hbp precursor [1, 2]. Substitution of these asparagines by a glycine and serine, respectively, prevents cleavage [3], denoted ***X***. Numbers displayed above the diagrams correspond to the amino acid positions of the wild-type Hbp precursor, calculated from the N-terminus. Insertion of a flexible linker (*FL*) comprising glycine and serine residues, is indicated. Insertion of mycobacterial antigens ESAT6 (*E-6*), Ag85B (*85*), $Ag85B_{[N]}$ (*85[N]*), $Ag85B_{[C]}$ (*85[C]*) and Rv2660c (*26*) as well as the insertion of chlamydial antigenic fragments $MOMP_{II}$ (*II*) and $MOMP_{IV}$ (*VI*). Also indicated is the insertion of the HA2stem (*HA*) and M2e (*M2e*) antigenic fragments, as well as the NP/PA/M1 T cell epitopes (*TC*) from influenza.

References

1. Dautin N, Barnard TJ, Anderson DE, Bernstein HD: Cleavage of a bacterial autotransporter by an evolutionarily convergent autocatalytic mechanism. *EMBO J* 2007, 26:1942-1952.
2. Roussel-Jazede V, Van Gelder P, Sijbrandi R, Rutten L, Otto BR, Luirink J, Gros P, Tommassen J, Van Ulsen P: Channel properties of the translocator domain of the autotransporter Hbp of Escherichia coli. *Mol Membr Biol* 2011, 28:158-170.
3. Jong WS, ten Hagen-Jongman CM, den Blaauwen T, Slotboom DJ, Tame JR, Wickstrom D, de Gier JW, Otto BR, Luirink J: Limited tolerance towards folded elements during secretion of the autotransporter Hbp. *Mol Microbiol* 2007, 63:1524-1536.
