# Supplementary material for: An autotransporter display platform for the development of multivalent recombinant bacterial vector vaccines
Source: Microb Cell Fact. 2014 Nov 25;13:162. doi: 10.1186/s12934-014-0162-8 (PMC4252983; doi:10.1186/s12934-014-0162-8)
Supplement: Additional file 3: Figure S3. — Expression of Hbp-Ag85B in degP- and dsbA-mutant strains. [file 12934_2014_162_MOESM3_ESM.pdf]

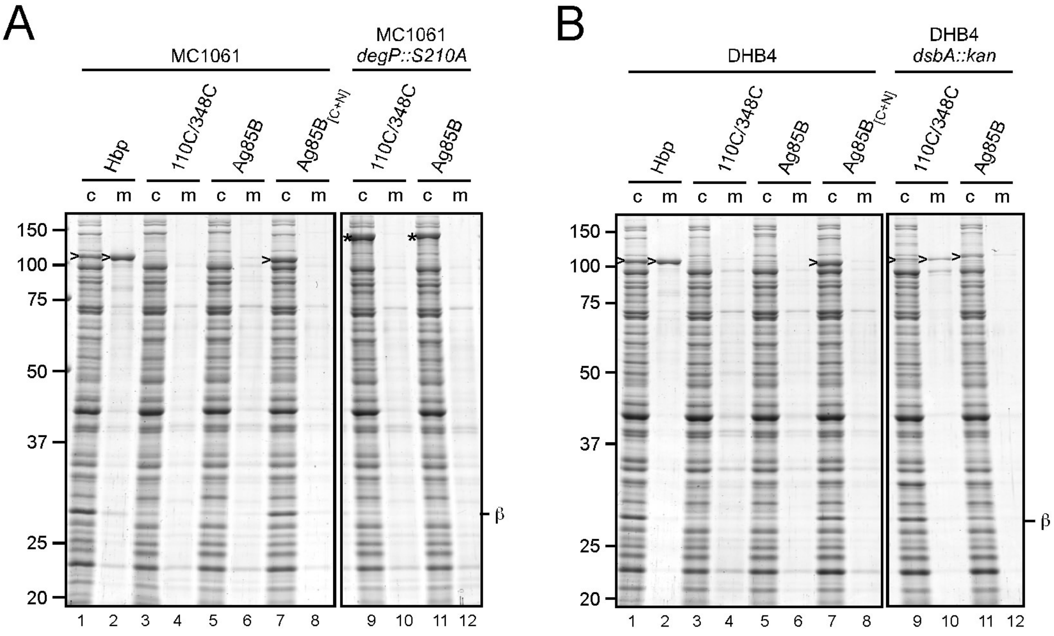


**Fig. S3. Expression of Hbp-Ag85B in *degP*- and *dsbA*-mutant strains. (A)** Expression of Hbp, Hbp110C/348C [1], Hbp(Δd1)-Ag85B and Hbp-Ag85B$_{[C+N]}$ in *E. coli* MC1061 and MC1061*degP::S210A* analyzed by SDS-PAGE and Coomassie staining as described in the legend to Fig. 2. In contrast to wild-type Hbp and Hbp-Ag85B$_{[C+N]}$, Hbp(Δd1)-Ag85B appears secretion incompetent and prone to degradation in wild-type *E. coli* MC1061, similar to secretion-incompetent Hbp110C/348C, which forms a secretion-blocking disulfide bond in the periplasm [1]. Expression of both secretion-incompetent Hbp variants is restored in strain MC1061*degP::S210A*, resulting in the accumulation of non-processed Hbp material. **(B)** Expression of Hbp, Hbp110C/348C, Hbp(Δd1)-Ag85B and Hbp-Ag85B$_{[C+N]}$ in *E. coli* DHB4 and DHB4*dsbA::kan* analyzed as described under *A*. In contrast to wild-type Hbp and Hbp-Ag85B$_{[C+N]}$, Hbp(Δd1)-Ag85B appears secretion incompetent and degraded in wild-type *E. coli* DHB4, similar to disulfide bond-forming mutant Hbp110C/348C. Whereas expression and secretion of Hbp110C/348C is substantially restored upon expression in strain DHB4*dsbA::kan* (also note elevated levels of cleaved β-domain), only a marginal improvement of expression and secretion is observed for Hbp(Δd1)-Ag85B. Cleaved Hbp passenger (>), non-cleaved Hbp species (*) and cleaved β-domain (β) are indicated. Molecular weight markers (kDa) are shown at the left side of the panels.

***References***

1. Jong WS, ten Hagen-Jongman CM, den Blaauwen T, Slotboom DJ, Tame JR, Wickstrom D, de Gier JW, Otto BR, Luirink J: Limited tolerance towards folded elements during secretion of the autotransporter Hbp. *Mol Microbiol* 2007, 63:1524-1536
