# Supplementary material for: An autotransporter display platform for the development of multivalent recombinant bacterial vector vaccines
Source: Microb Cell Fact. 2014 Nov 25;13:162. doi: 10.1186/s12934-014-0162-8 (PMC4252983; doi:10.1186/s12934-014-0162-8)
Supplement: Additional file 4: Figure S4. — Secretion of Ag85B[N] and Ag85B[C] upon fusion to Hbp. [file 12934_2014_162_MOESM4_ESM.pdf]

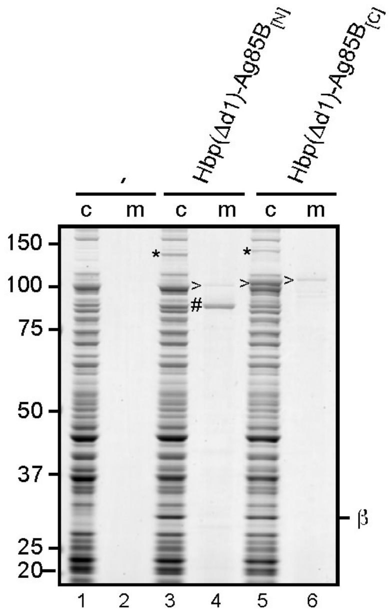

Fig. S4. Secretion of Ag85B$_{[N]}$ and Ag85B$_{[C]}$ upon fusion to Hbp. *E. coli* TOP10F' cells expressing either Hbp(Δd1)-Ag85B$_{[N]}$ or Hbp(Δd1)-Ag85B$_{[C]}$, or not expressing an Hbp variant (lanes 1-2) were analyzed by SDS-PAGE and Coomassie staining as described in the legend to Fig. 2. Cleaved Hbp passenger material (>), a proteolytic fragment of cleaved, released Hbp passenger material (#) and non-cleaved Hbp species (*) are indicated. Molecular weight markers (kDa) are shown at the left side of the panel.
