# Supplementary material for: An autotransporter display platform for the development of multivalent recombinant bacterial vector vaccines
Source: Microb Cell Fact. 2014 Nov 25;13:162. doi: 10.1186/s12934-014-0162-8 (PMC4252983; doi:10.1186/s12934-014-0162-8)
Supplement: Additional file 5: Figure S5. — Proteinase K accessibility of cleaved Hbp passenger-antigen fusions at the cell surface. [file 12934_2014_162_MOESM5_ESM.pdf]

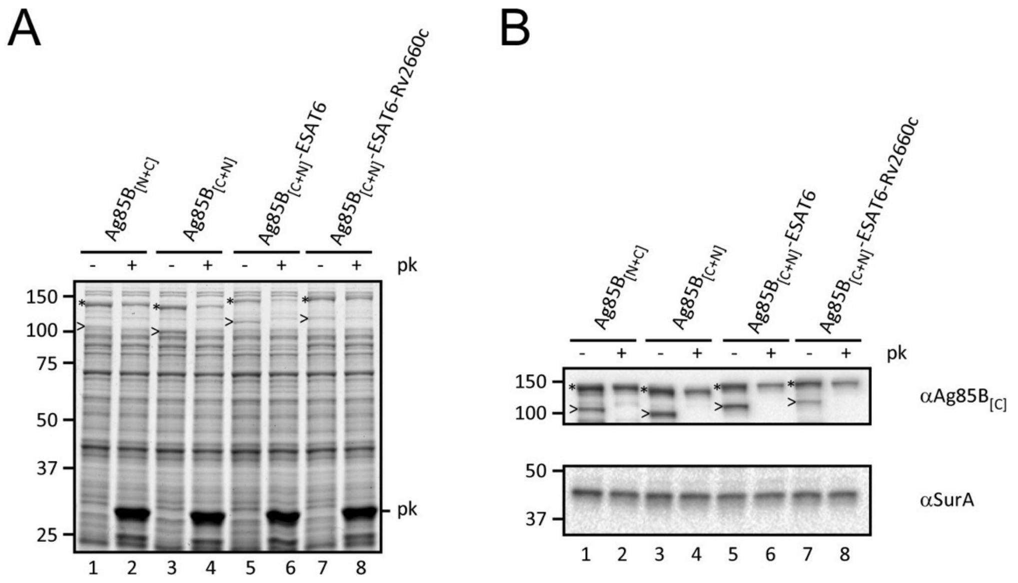

Fig. S5. Proteinase K accessibility of cleaved Hbp passenger-antigen fusions at the cell surface. **(A)** Cells expressing Hbp-Ag85B$_{[N+C]}$, Hbp-Ag85B$_{[C+N]}$, Hbp-Ag85B$_{[C+N]}$-ESAT6 or Hbp-Ag85B$_{[C+N]}$-ESAT6-Rv2660c described in the legend to Fig. 2 were collected by centrifugation and resuspended in icecold 50 mM Tris-HCl, PH 7.4, 1 mM CaCl. Subsequently, samples were incubated at 0°C for 30 min with (+) or without (-) Proteinase K (*pk*; 100 μg/ml). The reaction was stopped by addition of 0.1 mM PMSF and incubation on ice for 5 min. Samples were subjected to TCA precipitation before solubilization in SDS-PAGE sample buffer and analysis by SDS-PAGE and Coomassie staining. **(B)** Samples described under *A* were analyzed by immunoblotting using anti-Ag85B$_{[C]}$. As a control for cell integrity, samples were also analyzed using an antiserum against the periplasmic chaperone SurA, which is inaccessible for Proteinase K added to intact cells. Cleaved Hbp passenger (>) and non-cleaved Hbp species (*) are indicated. Molecular weight markers (kDa) are shown at the left side of the panels.
