# Supplementary material for: An autotransporter display platform for the development of multivalent recombinant bacterial vector vaccines
Source: Microb Cell Fact. 2014 Nov 25;13:162. doi: 10.1186/s12934-014-0162-8 (PMC4252983; doi:10.1186/s12934-014-0162-8)
Supplement: Additional file 6: Figure S6. — Secretion of ESAT6 inserted into d4. [file 12934_2014_162_MOESM6_ESM.pdf]

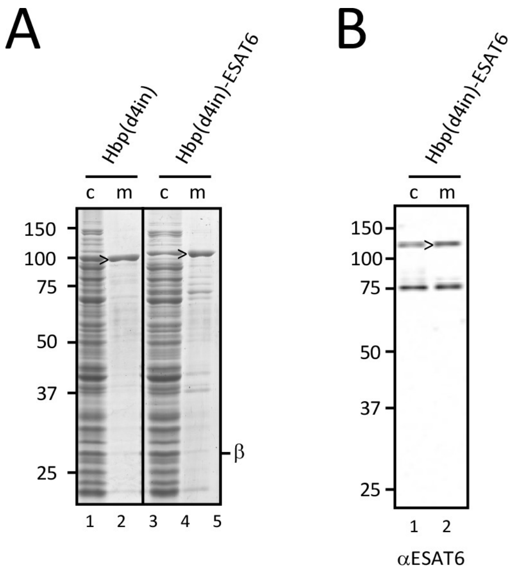

Fig. S6. Secretion of ESAT6 inserted into d4. (A) Secretion of Hbp(d4in) and Hbp(d4in)-ESAT6 analyzed by SDS-PAGE and Coomassie staining as described in the legend to Fig. 2. **(B)** Secretion of Hbp(d4in)-ESAT6 analyzed by immunoblotting using antibodies specific for ESAT6. Molecular mass (kDa) markers are indicated at the left side of the panels. Cleaved passengers (>) are indicated.
